# Contribution of ictal-interictal SPECT and SISCOM to preoperative localization of the epileptogenic zone in frontal lobe epilepsy

**DOI:** 10.1590/0100-3984.2026.0040

**Published:** 2026-09-27

**Authors:** Lauro Wichert-Ana, Ricardo Fernandes Amaral, Jaqueline Cardena Fernandes, Felipe Arriva Pitella, Felipe Mourato, Mery Kato, Tonicarlo Rodrigues Velasco, Frederico Nakane Nakano, Marina Teixeira Ramalho Pereira Dalio, Veriano Alexandre Jr, Ursula Thomé, Ana Paula Andrada Hamad, Antonio Carlos Santos, Américo Ceiki Sakamoto

**Affiliations:** 1 Nuclear Medicine and PET/CT Section, Department of Medical Imaging, Hematology and Oncology, Ribeirão Preto School of Medicine, University of São Paulo, Ribeirão Preto, SP, Brazil; 2 Epilepsy Surgery Center (CIREP) and Department of Neurosciences and Behavioral Sciences. Ribeirão Preto School of Medicine, University of São Paulo, Ribeirão Preto, SP, Brazil; 3 Bioengineering Interunits Program, São Carlos School of Engineering, University of São Paulo, São Carlos, SP, Brazil; 4 Nuclear Medicine Section, Hospital das Clínicas of the Federal University of Pernambuco. Recife, PE, Brazil

**Keywords:** Epilepsy, Epilepsy, frontal lobe, Seizures, Tomography, emission-computed, single-photon, Drug resistant epilepsy/surgery

## Abstract

**Objective:**

To describe the contributions of conventional single-photon emission computed tomography (SPECT) and subtraction ictal-interictal SPECT coregistered to magnetic resonance imaging (SISCOM) for the preoperative identification of the epileptogenic zone (EZ) in patients with frontal lobe epilepsy (FLE).

**Materials and methods:**

In this retrospective study, we reviewed medical records of all patients with refractory FLE who underwent frontal lobectomy at our institution between 2015 and 2020, all of whom had undergone at least one ictal SPECT, one interictal SPECT, one high-resolution magnetic resonance imaging examination, SISCOM, and at least one year of follow-up. Fourteen patients met the inclusion criteria.

**Results:**

After a mean follow-up of 32 months (range, 14–60 months), surgical outcomes were classified as good in five patients (35.7%) and poor in nine (64.3%). For localizing the EZ, SISCOM achieved the highest sensitivity (80%), negative predictive value (85%), and overall diagnostic accuracy (71%), whereas ictal SPECT had the highest specificity (70%) and interictal SPECT had the lowest performance overall.

**Conclusion:**

In patients with FLE, ictal and interictal SPECT alone do not seem to predict the surgical outcome. However, SISCOM seems to perform better for that purpose.

## INTRODUCTION

Epilepsy is a common chronic neurological disorder characterized by recurrent unprovoked seizures, with significant neurobiological, cognitive, and psychosocial consequences^**(^1^)**^. Frontal lobe epilepsy (FLE) represents a major diagnostic and therapeutic challenge in epilepsy surgery. Approximately one-third of all cases of epilepsy are refractory despite appropriate treatment with multiple antiseizure medications, and resective surgery may represent the only potentially curative option in selected cases^**(^2^,^3^)**^. However, surgical outcomes are generally less favorable in FLE than in temporal lobe epilepsy, with seizure-free rates being consistently lower in the former than in the latter^**(^4^,^5^)**^. Accurate preoperative localization of the epileptogenic zone (EZ) is therefore crucial. The complex corticocortical and subcortical connectivity of the frontal lobe facilitates rapid seizure propagation, often resulting in brief clinical manifestations and nonspecific scalp electroencephalographic (EEG) findings, which may hinder precise localization^**(^6^)**^. In addition, structural magnetic resonance imaging (MRI) may be normal or show only subtle abnormalities, particularly in cases of focal cortical dysplasia or cryptogenic epilepsy, further reducing the likelihood of favorable surgical outcomes^**(^7^,^8^)**^.

In this challenging clinical scenario, complementary functional imaging techniques have been increasingly investigated to improve localization accuracy and support surgical decision-making. Despite significant advances in preoperative diagnostic strategies, including interictal/ictal EEG, prolonged video-EEG monitoring, and hybrid positron emission tomography/computed tomography (PET/CT), the surgical management of nonlesional FLE continues to be particularly demanding^**(^9^)**^.

Brain perfusion single-photon emission computed tomography (SPECT) has emerged as a valuable complementary tool in the preoperative evaluation of drug-resistant epilepsy. Ictal SPECT may help localize the EZ by demonstrating focal hyperperfusion related to seizure onset, whereas interictal SPECT often reveals areas of relative hypoperfusion^**(^10^)**^.

When ictal and interictal SPECT findings are interpreted together, especially when subtraction ictal SPECT coregistered to MRI (SISCOM) analysis is employed, diagnostic accuracy may increase, allowing more reliable identification of the EZ than does visual analysis of isolated findings^**(^11^)**^. In addition, SISCOM may provide additional localization information in patients with inconclusive PET/CT findings or in those with persistent seizures after surgical resection. However, its specific role in predicting surgical outcomes in FLE has yet to be sufficiently established^**(^12^,^13^)**^. Given these challenges, the investigation of functional imaging techniques in FLE continues to be clinically relevant. Therefore, the aim of this exploratory study was to investigate the clinical contribution of conventional perfusion SPECT and SISCOM for preoperative localization of the EZ in patients with FLE.

## METHODS

### Subjects

This was a retrospective study of patients with medically refractory FLE who underwent resective surgery between 2015 and 2020 and had a complete preoperative evaluation—including ictal and interictal SPECT; high-resolution (1.5-T or 3.0-T) MRI; SISCOM processing; and at least one year of follow-up. Patients for whom the clinical or imaging data were incomplete were excluded, as were those who did not undergo surgical treatment and those with forms of epilepsy other than FLE. The patient selection process is summarized in Figure 1.

**Figure 1:**
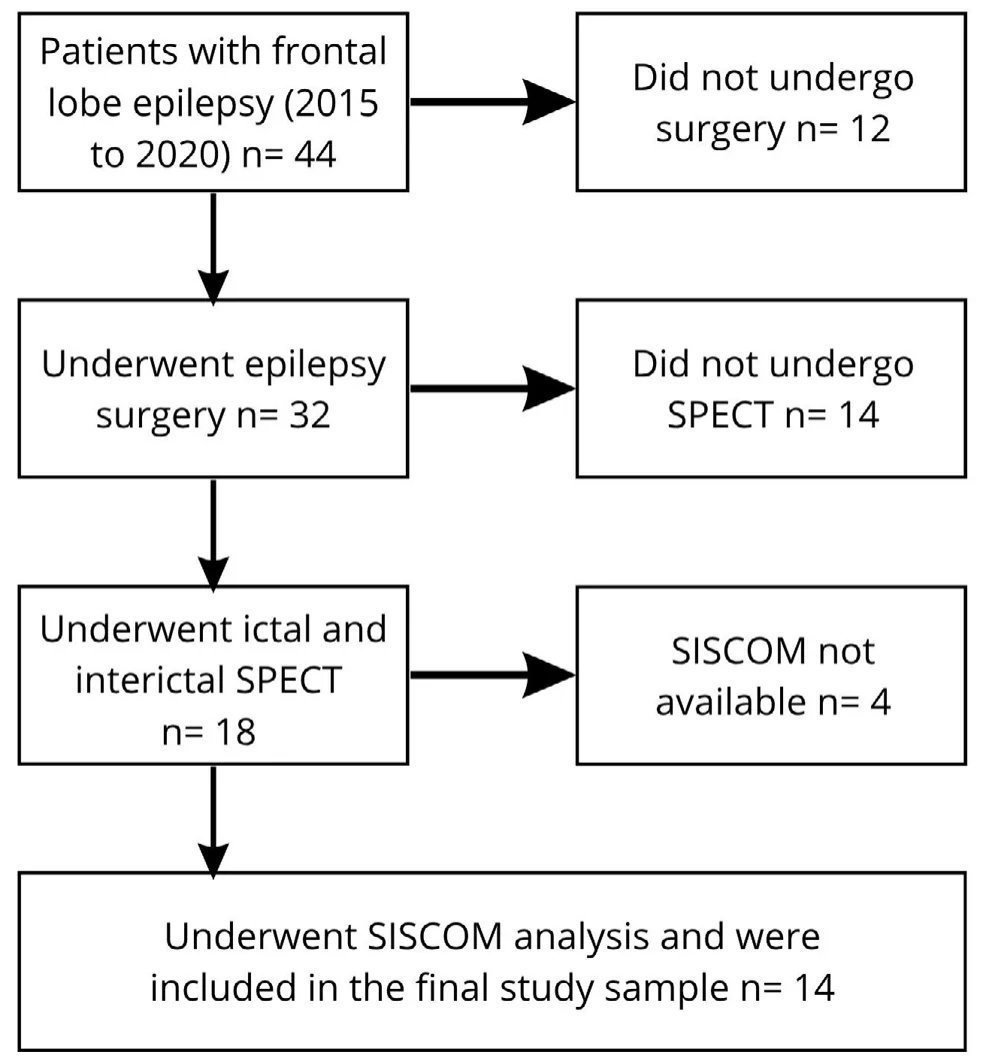
Flow chart of the patient selection process.

The following demographic and clinical data were collected: sex; current age; handedness; family history of neurological diseases; personal history of epilepsy; prenatal events; abnormal development; physical and neurological clinical findings; drug resistance; age at seizure onset; epileptic syndrome classification; seizure frequency; and type of seizures. The surgical data collected included age at surgery, area resected, postoperative MRI findings, histopathological findings of the resected tissue, follow-up period, and surgical outcome according to the Engel classification^**(^14^,^15^)**^. The local research ethics committee approved the study, and all participating patients gave written informed consent for the preoperative workup, including ictal and interictal SPECT.

### MRI acquisition and processing

High-definition MRI scans were acquired in a 1.5-T scanner (Magnetom Vision; Siemens Healthineers, Erlangen, Germany) or in a 3.0-T scanner (Achieva; Philips Medical Systems, Best, The Netherlands), in accordance with standardized epilepsy protocols. The imaging protocol included the following: a high-resolution sagittal T1-weighted sequence using a spoiled gradient recall technique; an axial turbo spin-echo sequence with dual-echo acquisition, providing proton density weighting and T2 weighting; a pair of fluid-attenuated inversion recovery sequences (one axial with 5-mm slices and one coronal with 3-mm slices), both oriented perpendicular to the hippocampal axis; a coronal short-tau inversion recovery sequence of the temporal lobes, acquired with thin, continuous 2-mm slices, also perpendicular to the hippocampus.

Although MRI findings were assessed using all MRI sequences in this epilepsy protocol, only the sagittal three-dimensional T1-weighted sequence was used for SISCOM processing. That MRI examination will hereafter be referred to as the preoperative MRI. The MRI performed after the surgery will be referred to as the postoperative MRI. The preoperative and postoperative MRIs were both used in our study.

Finally, MRI images were reviewed by two board-certified neuroradiologists with 30 years of practice, both of whom were blinded to all patient data. and, in case of discordance, a third neuroradiologist was consulted. Images were analyzed for structural abnormalities that could be related to epileptic seizures.

### Video-EEG monitoring

The video-EEG monitoring was reviewed by two board-certified neurophysiologists with more than 20 years of practice. Patients underwent continuous video-EEG monitoring on a digital platform (Vanguard Systems, Cleveland Clinic Foundation, Cleveland, OH, USA). Standard recordings employed a high-density electrode arrangement based on the 10-10 system, with additional sphenoidal electrodes placed bilaterally when clinically required. In selected cases, invasive techniques were used, including intraoperative electrocorticography and, when indicated, extraoperative subdural monitoring with strip or grid electrodes.

Interictal background rhythms and epileptiform discharges were reviewed in 5-min EEG segments sampled from each hour of the initial 24-h recording period, prior to the gradual tapering of antiepileptic medication to avoid withdrawal effects. The morphology and spatial distribution of interictal discharges were systematically evaluated. As described elsewhere^**(^16^)**^, a minimum of three seizures per patient was required and the temporal sequence of each ictal event—including the exact time of tracer injection—was assessed by board-certified clinical neurophysiologists.

### Ictal and interictal SPECT acquisition

All patients underwent ictal SPECT during video-EEG monitoring, followed by interictal SPECT a few weeks later. For the ictal studies, the radiotracer ethyl cysteinate dimer was administered at a maximum dose (in adults) of 1,295 MBq (35 mCi), immediately after the clinical or electrographic onset of a seizure. In children and adolescents, the administered activity was adjusted according to body weight. Interictal imaging was performed under resting conditions, in patients who had been seizure-free for at least 24 h, in a quiet, dimly lit room, without auditory or verbal stimulation. Patients kept their eyes open during image acquisition.

The SPECT scans were obtained with a dual-head rotating gamma camera (Philips BrightView XCT; Philips Healthcare, Cleveland, OH, USA) fitted with a low-energy, high-resolution collimator. Acquisition parameters included the following: photopeak centered at 140 keV with a 20% energy window, 64 projections per detector over 128° each, a 128 × 128 matrix, ×2 zoom, a total acquisition time of 30 min, and approximately 100,000 counts per projection per detector. The transaxial resolution was 7.9 mm at full width at half maximum, in accordance with National Electrical Manufacturers Association standards.

Image reconstruction was performed with the ordered-subset expectation maximization algorithm with three iterations and eight subsets, followed by the use of a second-order Butterworth filter (cutoff frequency, 0.3). Attenuation correction was achieved by using Chang’s first-order method (attenuation coefficient, 0.12 cm^−1^). The final SPECT voxel dimensions were 2.13 × 2.13 × 2.13 mm along the x, y, and z axes, respectively. The SPECT images were reviewed by two board-certified nuclear medicine physicians with 28 years of practice, both of whom were blinded to all patient data. In cases of discordance, a third nuclear medicine physician with 16 years of practice was consulted.

### SISCOM processing

Developed by researchers at the Mayo Clinic, SISCOM is a computer-assisted diagnosis technique. For SISCOM analysis, ictal SPECT is subtracted from interictal SPECT, and the result is registered to the MRI, yielding a functional map of the brain that highlights the areas most active during seizures and correlates with MRI structural information.

In the present study, SISCOM was performed with ANALYZE software, version 10.0 (AnalyzeDirect, Overland Park, KS, USA). An automatic algorithm based on voxel similarity, quantified by the mutual information metric, was used in order to register SPECT–MRI and MRI–MRI pairs. The specialist could manually adjust the image size, orientation, and position to achieve more precise registration.

Before SISCOM is performed, the brain is segmented from a preoperative MRI by using morphological and manual thresholding techniques. The SISCOM processing protocol began by importing ictal and interictal SPECT images, as well as preoperative and postoperative MRI scans, into the working environment of the software. The MRI scans were resized during importation, with all voxel dimensions set to 1.0 mm to achieve spatial isotropy. The SPECT images were flipped vertically.

The SISCOM workflow was as follows: the ictal SPECT was registered to the preoperative MRI; the interictal SPECT was registered to the preoperative MRI; the SPECT images were manually filtered by threshold segmentation to extract brain activity from scattering noise and scalp activity; the radiopharmaceutical uptake level was normalized in the SPECT images; the ictal SPECT was subtracted from the interictal SPECT; the mean value and standard deviation were calculated for the result of the subtraction, and all voxels with a value less than two times the standard deviation above the mean were ignored; the filtered result was then registered to the preoperative MRI; finally, as an additional step in our SISCOM protocol, the filtered subtraction map was registered to the postoperative MRI.

### Evaluation of SPECT and SISCOM images

The SISCOM analysis was performed retrospectively, for research purposes only, and did not influence surgical decision-making or the extent of resection. Conventional SPECT scans were reviewed in a blinded fashion by two experienced examiners, working independently. The examiners focused on detecting regions of cerebral hyperperfusion in ictal studies and hypoperfusion in interictal studies. Visual grading was applied to categorize the findings as mild, moderate, or marked. If the primary reviewers disagreed, a third specialist provided the final assessment. Similarly, SISCOM images were analyzed, also in a blinded fashion, by the same experts. Areas of abnormality were ranked by relative intensity, from most to least prominent.

As the gold standard for the correct localization of the EZ, we considered the set of findings from the preoperative evaluation that informed the surgical site selection. We registered each SPECT and SISCOM with its corresponding postoperative MRI to assess the contribution of each functional technique to the localization of the EZ.

The presumed EZ (PEZ) location was considered the site of the most pronounced finding in SPECT, whereas it was considered the site of the most intense finding in SISCOM. In the first qualitative analysis, the results of ictal and interictal SPECT scans and of the SISCOM analysis were compared with the area of the cerebral cortex resected (standard EZ location).

Figure 2 proposes a classification of SISCOM findings in the operated frontal lobes of patients with FLE. If the resected area was found to be at the same site as the result of the SISCOM, it was primarily classified as coincident and secondarily classified as coincident included if the resected area fully included the SISCOM finding or if the SISCOM finding was on the border of the resected area. If the SISCOM finding was outside but very close to the resected area, it was classified as coincident excluded. When the resected area was far from the SISCOM finding, it was classified as noncoincident—either ipsilateral, contralateral to the hemisphere of the resection, or bilateral. Multiple or inconclusive SISCOM findings were also classified as noncoincident.

**Figure 2:**
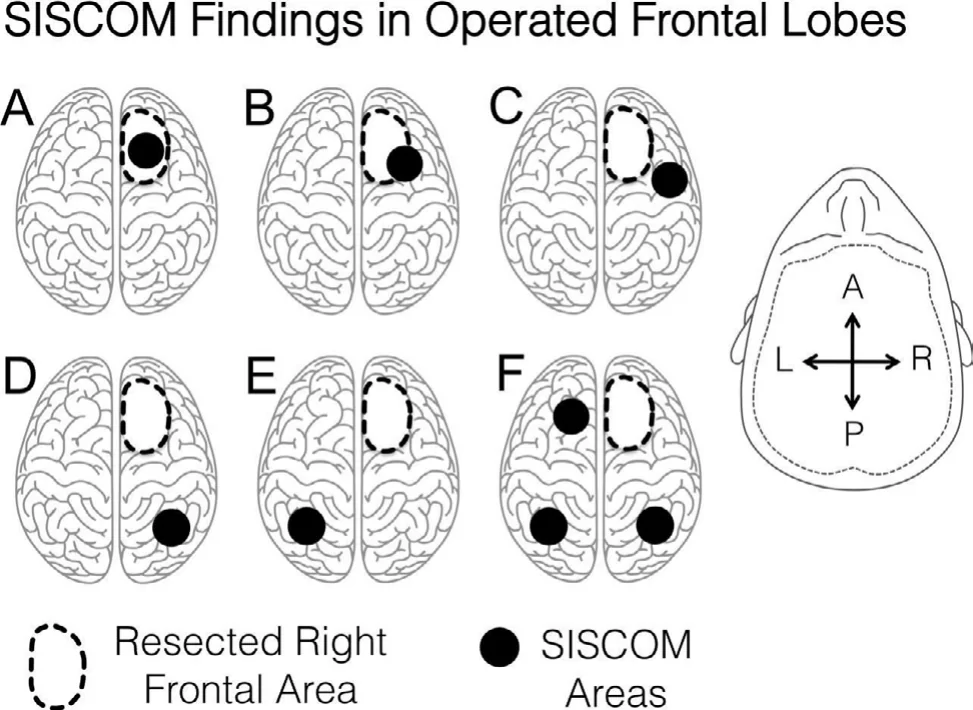
Proposed classification of SISCOM findings in operated frontal lobes of patients with FLE: coincident SISCOM localization, included in the resected cortex (**A**), partially excluded from the resected cortex (**B**); or entirely excluded from but close to the resected cortex (**C**); and noncoincident SISCOM localization, ipsilateral to the resected cortex (**D**), contralateral to the resected cortex (**E**); or with multiple hotspots (**F**).

In a second quantitative analysis, we measured performance parameters, including sensitivity, specificity, positive predictive value, negative predictive value, and overall accuracy. The SPECT and SISCOM results were classified as coincident (either included in or excluded from the resected area) or noncoincident (far from the resected area or inconclusive). In cases in which the result was coincident (included or excluded), it was considered a true positive if the patient had a good surgical outcome (Engel class I or II) and a false positive if the patient had a poor surgical outcome (Engel class III or IV). Similarly, when the result was classified as noncoincident or inconclusive, it was considered a false negative if the surgical outcome was good and a true negative if it was poor. Performance was assessed through the use of the following formulas:


$$\begin{array}{l} Sensitivity=true\;positives/\left[true\;positives+false\;negatives\right]\\ Specificity=true\;negatives/\left[true\;negatives+false\;positives\right]\\ Positive\;predictive\;value=true\;positives/\left[true\;positives+false\;positives\right]\\ Negative\;predictive\;value=true\;negatives/\left[\text{true }negatives+\;negatives\right]\\ Accuracy=\left[true\;positives+true\;negatives\right]/\left[true\;positives+false\;positives+false\;negatives+true\;negatives\right] \end{array}$$

## RESULTS

### Demographic data

Table 1 describes the demographic, clinical, and surgical characteristics of the patient sample. A total of 14 patients met the inclusion criteria and underwent epilepsy surgery. The mean age at epilepsy onset was 6.7 years (range, 0–17 years), and the mean age at evaluation was 21.4 years (range, 6–46 years). Five patients were female, and nine were male. Physical and neurological examinations were normal in four (28.6%) of the patients and abnormal in ten (71.4%). The number of seizures per month ranged from four (average of one seizure/week) to 600 (average of 20 seizures/day). After a mean follow-up of 32 months (range, 14–60 months), the surgical outcome was categorized as good (Engel class I or II) in five (35.7%) of the patients and as poor (Engel class III or IV) in nine (64.3%).

**Table 1:** Demographic, clinical, and surgical data.

Patient	Age (years)/Sex/Handedness	Physical and neurological status	Age at epilepsy onset	Seizure type(s)	Seizures per month	MRI findings	Type of surgery	Histopathology	Outcome (follow-up in months)
			(years)						
1	30/M/R	Facial diplegia	5.0	FS; FSIC; SGTCS	45	L frontal tumor	L frontal resection, anterior callosotomy	Ganglioglioma (WHO grade I)	IVa (60)
2	16/F/R	Cognitive delay	1.6	FSIC during sleep; SGTCS	30	Normal	L frontal anterior and inferior disconnection, anterior callosotomy	Subcortical heterotopia	IVa (32)
3	9/M/R	Cognitive delay	2.1	L body GTCS during sleep	30	R medial precentral frontal FCD	R inferior frontal resection (sparing hand motor area)	FCD Ib (Palmini)	IVb (16)
4	15/M/L	Bradypsychism	0.9	FSIC; SGTCS	45	L frontal temporal atrophy	L frontal partial lobectomy	Astrogliosis, spongiosis, microcysts in the neuropil	IVb (24)
5	30/M/R	Facial scars, bradypsychism, hypomimia	3.0	Lennox–Gastaut syndrome; TS	30	R frontal venous angioma	R frontal lobectomy, anterior callosotomy	Vascular malformation angiomatosis	IVa (48)
6	25/F/R	Cognitive delay	8.0	FSIC; SGTCS	30	Normal	R frontal lobectomy (sparing motor area)	Cortical microdysgenesis (grade I)	IIIa (24)
7	20/M/R	Normal	9.0	Scream; R face and upper limb clonus; rare SGTCS	10	L frontal low-grade tumor	L frontal lesion resection	DNT (WHO grade I)	Ia (54)
8	32/M/R	R hemiparesis	13.0	FS; R body cold sensation; R lower limb clonus; preserved consciousness	600	L parietal dysplasia; L mesial temporal sclerosis	L supplementary motor frontal area resection	FCD (Palmini type Ia)	IIIa (27)
9	6/F/R	Normal	0.6	FS; L foot clonus; Jacksonian march; L body SGTCS	4	R frontal motor lesion (tumor or dysplasia)	R superior frontal resection	Oligoastrocytoma; glioma grade II	Ia (39)
10	46/F/R	Normal	17.0	FS; FSIC; rare SGTCS	30	R frontal FCD	R superior medial frontal resection	FCD (Palmini type IIb)	Ia (14)
11	20/F/R	Normal	1.5	FS; FSIC; L body clonus	150	R anterior insula lesion (tumor or dysplasia)	R medial inferior orbital frontal and R anterior insula resection	FCD (Palmini type IIb)	Ib (25)
12	20/M/L	R upper limb paresis, speech sound disorder, speech delay	1.0	FS R upper limb clonus; SGTCS	30	L parietal atrophy and gliosis	L superior medial parietal multiple subpial transection	Reactive astrogliosis	IVb (38)
13	19/M/L	Bradypsychism	14.0	FS; FSIC; rapid recovery of consciousness	60	L frontal vascular malformation	L superior medial frontal resection (sparing motor area)	Ganglioglioma	Ia (24)
14	11/M/R	Cognitive delay	0.2	TS	60	Tuberous sclerosis (multiple tubers); subependymal giant cell astrocytoma	L superior medial frontal	Tuber, astrocytoma	IVb (15)

M, male; F, female; R, right; L, left; FS, focal seizure (without impaired consciousness); FSIC, focal seizure with impaired consciousness; WHO, World Health Organization; TS, tonic seizure; GTCS, generalized tonic–clonic seizure; SGTCS, secondary GTCS; FCD, focal cortical dysplasia; Ia, Engel class characterized by being completely seizure-free, with no auras or seizures; Ib, Engel class characterized by nondisabling simple partial seizures only; IIIa, Engel class characterized by worthwhile improvement with prolonged seizure reduction, but less than a 2-year complete block; IVa, Engel class characterized by no worthwhile improvement, with only a brief or insufficient reduction in seizure frequency; IVb, Engel class characterized by no improvement at all or a persistent, unchanged seizure frequency.

### Structural neuroimaging

Table 1 presents a set of overlapping abnormalities identified on structural neuroimaging. The results of the examinations were focal cortical dysplasia (FCD) in two patients (14.3%), tumors in two (14.3%), nonspecific findings (either FCD or tumor) in two (14.3%), vascular malformations in two (14.3%), mesial temporal sclerosis in one (7.1%), tuberous sclerosis in one (7.1%), cortical atrophy in one (7.1%), and normal in two (14.3%).

Comparing MRI findings with the surgical outcomes from ten patients with suspected epileptogenic lesions (patients 1, 3, 4, 5, 7, 9, 10, 11, 12, and 13), we found that five of those patients had good surgical outcomes and five had poor ones. Both of the patients with inconclusive MRI findings (multiple lesions, patients 8 and 14) had poor surgical outcomes, as did both of the patients with normal MRI findings (patients 2 and 6).

### Video-EEG monitoring

Table 2 describes the clinical and electroencephalographic (video-EEG) details of the seizures. The ictal EEG showed some localized findings in eight (57.1%) of the patients (patients 2, 3, 4, 6, 8, 10, 11, and 14), lateralized findings in one (7.1%, patient 1), and nonlocalizing, nonlateralizing, or normal findings in five (35.8%, patients 5, 7, 9, 12, and 13). The interictal EEG showed some localized findings in nine (64.3%) of the patients (patients 1, 3, 4, 5, 6, 7, 9, 10, and 11), lateralized findings in three (21.4%, patients 2, 8, and 13), and nonlocalizing, nonlateralizing, or normal findings in two (14.3%, patients 12 and 14). Six patients (42.9%) underwent invasive EEG monitoring, and localization of the EZ was improved in only one (16.7%) of those patients; the findings in the remaining five patients were the same as those obtained with noninvasive EEG.

**Table 2:** Video-EEG and neuroimaging data.

Patient	Ictal semiology findings	Ictal EEG findings	Interictal EEG findings	EZ localization	Seizure duration	Injection delay	Contributions		
					(s)	(s)	Ictal SPECT	Interictal SPECT	SISCOM
1	FSIC; contraction of upper limbs; confusion	L slow spike-wave and recruiting rhythm	Normal BA, L predominating BL frontal slow spike-wave activity	L frontal	45	53	INC	CO-IN	CO-EX
2	During sleep; FS; tonic; hypermotor seizures	L frontal temporal theta rhythm	Normal BA; L frontal, temporal, and occipital spikes	L frontal	54	25	NCO	INC	NCO
3	Eyelid blinking; L face clonic contraction; TS of upper limbs	R frontal motor and parietal spikes	Normal BA; R frontal motor and parietal spike-waves and sharp waves	R frontal motor	26	26	CO-EX	NCO	CO-EX
4	FSIC; dysarthria; R-hand dystonia; automatisms; confusion	L theta/delta activity, predominating in L temporal lobe	Normal BA; L frontal motor spikes and sharp waves	L hemisphere	58	34	NCO	CO-IN	NCO
5	Face clonus; TS; abduction of upper limbs; L ocular deviation	Midline spikes; polyspikes; diffuse recruiting rhythm	Abnormal BA; R frontal slow waves; midline spikes; BL synchrony	R medial frontal	27	25	NCO	INC	NCO
6	TS; hyperkinetic automatisms of lower limbs	R frontal rhythmic activity; diffuse recruiting rhythm	R frontal spikes	R frontal	29	29	CO-IN	INC	NCO
7	Scream; R face and upper limb clonus; GTCS	Diffuse sharp waves and theta/delta activity	Normal BA; L frontal motor spikes; spike-wave complex	L frontal motor	63	21	NCO	CO-IN	CO-EX
8	L body cold or burning sensation; R lower limb clonus	L desynchronization; L frontal-temporal theta/delta slow activity	Abnormal BA in the L hemisphere	L frontal medial	47	26	NCO	CO-IN	CO-IN
9	FSIC; staring; L foot clonus	Diffuse theta/delta activity	Normal BA; R frontal motor spikes	R frontal motor	36	24	CO-IN	INC	CO-IN
10	Brief FSIC; automatisms; grimace	R frontal temporal repetitive discharges	Normal BA; R frontal delta activity	R frontal	51	25	CO-IN	INC	CO-IN
11	FSIC during sleep; L hand dystonia; L leg extension	BL theta/delta activity, late R temporal rhythmic activity	Abnormal BA; R frontal temporal spikes; and slow wave activity	R frontal temporal	27	40	NCO	INC	NCO
12	TS; R tonic-clonic seizure	No ictal EEG finding	L hemisphere continuous spikes; R frontal parietal temporal spikes	L frontal middle inferior	61	24	INC	NCO	NCO
13	FSIC; automatisms	Diffuse beta activity	Normal BA; L hemisphere showing sharp, slow-wave spikes	L hemisphere	26	23	NCO	CO-IN	CO-IN
14	TS	Diffuse sharp waves, BL parietal occipital beta activity	Abnormal BA; multifocal irregular spikes; polyspikes; and sharp waves	BL posterior cortex	63	24	NCO	INC	NCO

R, right; L, left; BL, bilateral; BA, background activity; CO, coincident; IN, included; EX, excluded; NCO, noncoincident; INC, inconclusive; FSIC, focal seizure with impaired consciousness; TS, tonic seizure.

### Perfusion SPECT and SISCOM findings

Table 2 describes the SPECT and SISCOM findings. The radiotracer injection was ictal in 10 patients (71.4%, patients 2, 4, 5, 7, 8, 9, 10, 12, 13, and 14) and peri-ictal in four (28.6%, patients 1, 3, 6, and 11). The mean seizure duration was 43.8 s (range, 6–63 s), and the mean interval between seizure onset and injection was 28.5 s (range, 29–53 s).

In four (28.6%) of the ictal SPECT scans, the findings were coincident with the surgical resection site (three included in and one external to the resected area), whereas they were noncoincident in eight (57.1%) and inconclusive in two (14.3%). In five (35.7%) of the interictal SPECT scans, the findings were coincident with the surgical resection site (all included in the resected area), whereas they were noncoincident in two (14.3%) and inconclusive in seven (50.0%). In seven (50.0%) of the SISCOM analyses, the findings were coincident with the surgical resection site (four included in and three external to the resected area), whereas they were noncoincident in seven (50.0%). Therefore, SISCOM outperformed ictal and interictal SPECT in terms of the proportion of coincident findings (50.0% vs. 28.6% and 35.7%, respectively).

There was no association between better surgical outcomes (Engel class I or II) and coincident findings or between poor outcomes (Engel class III or IV) and noncoincident/inconclusive findings for ictal SPECT findings (chi-square = 0.49, p = 0.48) or interictal SPECT findings (chi-square = 0.06, p = 0.80). However, there was a slight trend toward an association for SISCOM findings (chi-square = 2.38, p = 0.094). Figure 3 illustrates examples of SISCOM findings classified as coincident included and coincident excluded.

**Figure 3:**
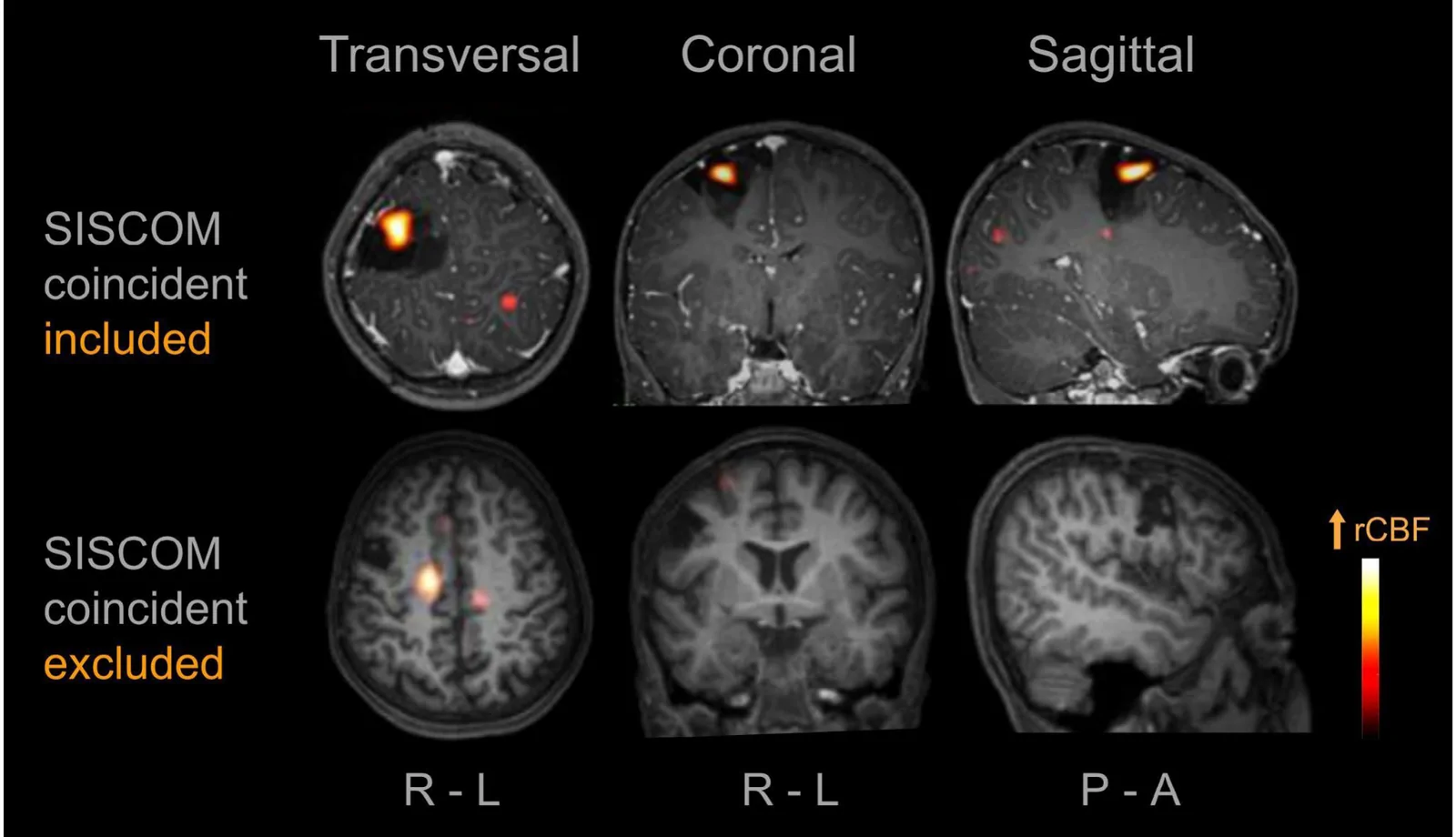
Two examples of coincident SISCOM findings—included and excluded. The first row shows transversal, coronal, and sagittal views of the resection site, including the SISCOM finding. The second row shows the SISCOM finding excluded but close to the resection area.

## DISCUSSION

The present study evaluated the contributions of ictal-interictal SPECT and SISCOM to the localization of the EZ in patients with FLE. The SISCOM analysis exhibited performance superior to that of conventional ictal and interictal SPECT alone, with the highest sensitivity, negative predictive value, and overall diagnostic accuracy in determining the location of the EZ (80%, 85%, and 71%, respectively). For that purpose, ictal SPECT had the highest specificity (70%) and interictal SPECT had the lowest performance on most parameters. In addition, there was a trend toward SISCOM predicting better seizure control outcomes. However, this was an exploratory study based on a small sample of patients. There is a need for studies involving larger patient samples, in order to evaluate the contribution of SISCOM, mainly in patients with FLE and nonspecific or normal findings on high-resolution MRI. Although SISCOM demonstrated higher diagnostic performance, that did not translate into a clear association with favorable surgical outcomes^**(^17^)**^. This apparent dissociation may reflect the inherent complexity of FLE, in which the EZ may not correspond to a single focal region but rather to a distributed epileptogenic network^**(^18^)**^. Therefore, even accurate localization of the region of maximal ictal perfusion may not guarantee complete resection of the epileptogenic network, particularly in cases of rapid seizure propagation or involvement of eloquent cortex, which can limit the extent of epilepsy surgery^**(^19^)**^. In addition, using the resection site as a surrogate gold standard may introduce bias, given that it does not necessarily reflect the true EZ, especially in patients who subsequently have suboptimal postoperative outcomes.

Epilepsy surgery for FLE has been the subject of several clinical investigations. One study showed that video-EEG locates the EZ in up to 90% of cases of drug-resistant focal epilepsy, and 61.4% of the patients in that study underwent invasive video-EEG^**(^11^)**^. Another study showed that, at postoperative follow-up, 68.1% of patients with FLE had evolved to Engel class I, whereas 11.4% had evolved to Engel class II, 9.0% had evolved to Engel class III, and 11.4% had evolved to Engel class IV^**(^20^)**^. Yet another study evaluated patients who underwent resective surgery for FLE using invasive video-EEG and found that 55.0% of cases were lesional and 45.0% were nonlesional^**(^21^)**^. After a 79-month follow-up period, the surgical outcomes were categorized as Engel class I in 57.0% of the patients in that study, as Engel class II in 16.0%, and as unsatisfactory in 27.6%. Approximately 65% of patients with FLE have a good surgical outcome^**(^22^)**^. An additional factor that may have influenced the performance of ictal SPECT is the timing of the radiotracer injection. In FLE, seizures are often brief and characterized by rapid propagation, increasing the likelihood that tracer injection occurs after the spread of a seizure rather than at its onset, given that tracer uptake reflects cerebral perfusion at the time of its arrival in the brain and is highly dependent on the timing of the injection^**(^23^)**^. Consequently, ictal SPECT may reflect propagation pathways instead of the true EZ, which could partially explain its lower sensitivity and limited concordance with surgical outcomes in our cohort^**(^24^)**^.

Our findings differ from those described in the literature. In our cohort, the proportion of seizure-free patients was relatively low (35.7%), possibly due to selection bias. Our study cohort comprised patients referred for surgery after inconclusive or conflicting preoperative findings on noninvasive examinations, which may not represent the broader population of patients with FLE. Only patients who required ictal and interictal SPECT were included in our present study to elucidate the location of the EZ because of nonspecific video-EEG findings, inaccurate MRI findings, or findings not in agreement with the video-EEG. Therefore, our patients presented with significant heterogeneity in the possible causes of FLE, including FCD, tuberous sclerosis, tumors, and other etiologies. From a clinical perspective, the SISCOM findings should be interpreted within a multimodal preoperative framework. Its main contribution appears to be in cases with inconclusive or discordant findings from conventional investigations, such as nonlocalizing MRI or ambiguous video-EEG data. In such scenarios, SISCOM may provide complementary spatial information that supports hypothesis generation regarding the EZ. However, it should not be considered in isolation, because its findings must be integrated with electroclinical and structural data to guide surgical decision-making^**(^25^)**^.

Contributions from ictal SPECT, interictal SPECT, and SISCOM have been used in order to localize the EZ. A previous study evaluated the ability of SPECT, SISCOM, and PET to identify the EZ in a group of 16 patients with refractory epilepsy^**(^26^)**^. The combined performance of the three examinations (PET, SPECT, and SISCOM) showed a good diagnostic yield, identifying the EZ in 87% of patients. The authors also showed that SISCOM assists the SPECT analysis, increasing the sensitivity of the perfusion study. The marked heterogeneity of underlying etiologies in our cohort may also have influenced the performance of the imaging modalities evaluated. Different pathological substrates may present distinct patterns of perfusion and metabolic abnormalities, potentially affecting the sensitivity of SPECT and SISCOM, because variations in underlying pathology can alter the spatial extent and characteristics of functional imaging abnormalities^**(^27^)**^. This variability further underscores the need for individualized interpretation of imaging findings within the broader clinical and neurophysiological context. Another study analyzed the effectiveness of SISCOM in localizing the EZ in 44 children who were candidates for resective surgery^**(^28^)**^. In that study, the SISCOM location was compared with the PEZ determined by video-EEG, and the PEZ was localized in 51.0% of patients, with lateralization being observed in 17.6%. The SISCOM showed better localizations than did ictal SPECT, ^18^F-fluorodeoxyglucose PET, and MRI alone. The SISCOM was also helpful in locating the PEZ in 25% of patients in whom it was poorly located by video-EEG. The authors concluded that SISCOM is a reliable tool for locating the EZ in clinical practice, especially in patients with nonlocalizing MRI or poorly localizing video-EEG.

Finally, discordant findings between the SISCOM analyses and surgical outcomes highlight the complexity of preoperative evaluation in FLE and the limitations of relying on a single imaging modality. Noncoincident results in patients with favorable outcomes may reflect successful resections guided by other modalities, whereas coincident findings associated with poor outcomes may indicate incomplete resection or involvement of a broader epileptogenic network. In this context, the relatively high negative predictive value of SISCOM suggests a potential role in identifying patients less likely to benefit from surgery, although this requires further validation. In addition, although structural MRI remains essential, its limitations in cases with subtle or nonlocalizing abnormalities underscore the added value of SISCOM as a complementary tool. Recent studies have emphasized that no single imaging modality is sufficient for accurate localization and that multimodal integration provides superior diagnostic performance^**(^29^,^30^)**^. Furthermore, variability in SISCOM findings, including the inclusion of propagation-related regions, further supports the need for cautious, integrated interpretation^**(^10^)**^. Moreover, SISCOM appears to be particularly useful in patients for whom conventional investigations produce inconclusive results, although its role in surgical decision-making and outcome prediction should be further explored in larger studies

### Limitations of the study

Some limitations of this study should be acknowledged. First, this was a retrospective analysis conducted at a single tertiary-care center, and the number of patients included was relatively small. As a consequence, the statistical power is limited, and the results should be interpreted with caution, particularly regarding their broader applicability. Another important aspect concerns patient selection. Only individuals with inconclusive or discordant findings on conventional noninvasive preoperative investigations, such as EEG and MRI, were included. Although this reflects a common, clinically challenging scenario in epilepsy surgery programs, it may have introduced a degree of selection bias and could partly explain the relatively unfavorable surgical outcomes observed in our cohort. In addition, the underlying structural abnormalities were heterogeneous, including focal cortical dysplasia, tuberous sclerosis, and tumor-related lesions. That variability makes it more difficult to interpret imaging performance consistently and limits the ability to establish clear correlations between SISCOM findings and specific pathological substrates. Therefore, the results of the present study should be regarded as exploratory. Further studies involving larger, more homogeneous patient populations, preferably prospective studies conducted in multicenter settings, are warranted in order to better define the role of SISCOM in the preoperative evaluation of FLE.

## CONCLUSIONS

Conventional ictal and interictal SPECT, employed in isolation, do not seem to be capable of accurately predicting surgical outcomes. Although SISCOM demonstrated higher sensitivity, negative predictive value, and accuracy for EZ localization, as well as showing a trend toward predicting surgical prognosis, those findings should be interpreted with caution. Given the small sample size, the prognostic value of SISCOM cannot be unequivocally established, and further studies with larger cohorts are needed in order to confirm its clinical utility.

## Data Availability

The research data are available within the document.
